# Carbohydrate Metabolism and Diabetes

**Published:** 1906-12

**Authors:** 


### Reviews of Books.

**Carbohydrate Metabolism and Diabetes.** By F. W. Pavy, M.D. Pp. xi., 138. London: J. and A. Churchill. 1906.

In this interesting book Dr. Pavy views carbohydrate metabolism from the physiological and pathological aspects. One of the main "arguments" in the book is that the glycogenic doctrine having been founded on erroneous data should be abandoned, particularly as this doctrine leads to serious error in practice. Dr. Pavy advances his own views, which are based on a very considerable amount of experimental observation and clinical experience, with much force and lucidity. If sugar circulates as such in the blood it is excreted by the kidneys, for sugar cannot exist in simple solution in the blood without the appearance of glycosuria. He has shown that sugar contained in the blood is not only present in the form of glucose, but that after hydrolysis an additional quantity makes its appearance. He maintains that this phenomena is explained either by the presence of carbohydrate matter of lower cupric oxide power than glucose, or by the liberation of sugar from a glucoside. Proteid matter has recently been shown to be of a glucoside character, and to be able to yield cleavage carbohydrate. Forming a constituent part of a large proteid molecule, the carbohydrate element is not discharged by the kidneys. Microscopical examination of the villi of the small intestine during digestion reveals a very largely increased number of lymphocytes within the villi, and it is known that a well-marked lymphocytosis occurs in the blood after a meal. This implies an increase of proteid carried to the blood. Pavy regards these lymphocytes as carriers of food from the intestine, and suggests that the sugar is carried as a side chain in the proteid molecule, from which it can be removed when required by the tissues. In the simplest form of diabetes, which he calls "alimentary," some of the sugar fails to be properly assimilated by the proteid element in the lymphocytes, and then entering the blood in the free state is filtered off by the kidneys. In the more serious variety, which he terms "composite," to the faulty anabolism is added a faulty katabolism attended with the liberation of sugar. In this latter variety the acetone series of products are also formed. He considers as a point of great importance the fact that sugar circulating in the blood acts as an extremely toxic substance to the bodily tissues, and compares its action with alcohol and other well-known toxins. Moreover, by its powerful diuretic action it leaves the body defective in fluid. The rational treatment of the disease, which contains many points of interest, and which is based on the principles enunciated in the work, forms the latter part of the bock.

